# Mechanisms of oxidative stress and alterations in gene expression by Libby six-mix in human mesothelial cells

**DOI:** 10.1186/1743-8977-7-26

**Published:** 2010-09-11

**Authors:** Jedd M Hillegass, Arti Shukla, Maximilian B MacPherson, Sherrill A Lathrop, Vlada Alexeeva, Timothy N Perkins, Albert van der Vliet, Pamela M Vacek, Mickey E Gunter, Brooke T Mossman

**Affiliations:** 1 Department of Pathology, University of Vermont College of Medicine, 89 Beaumont Avenue, Burlington, VT 05405, USA; 2 Department of Medical Biostatistics, University of Vermont College of Medicine, 25 Carrigan Avenue, Burlington, VT 05405, USA; 3 Department of Geological Sciences, University of Idaho, PO Box 443022, Moscow, ID 83843, USA

## Abstract

**Background:**

Exposures to an amphibole fiber in Libby, Montana cause increases in malignant mesothelioma (MM), a tumor of the pleural and peritoneal cavities with a poor prognosis. Affymetrix microarray/GeneSifter analysis was used to determine alterations in gene expression of a human mesothelial cell line (LP9/TERT-1) by a non-toxic concentration (15×10^6 ^μm^2^/cm^2^) of unprocessed Libby six-mix and negative (glass beads) and positive (crocidolite asbestos) controls. Because manganese superoxide dismutase (MnSOD; SOD2) was the only gene upregulated significantly (p < 0.05) at both 8 and 24 h, we measured SOD protein and activity, oxidative stress and glutathione (GSH) levels to better understand oxidative events after exposure to non-toxic (15×10^6 ^μm^2^/cm^2^) and toxic concentrations (75×10^6 ^μm^2^/cm^2^) of Libby six-mix.

**Results:**

Exposure to 15×10^6 ^μm^2^/cm^2 ^Libby six-mix elicited significant (p < 0.05) upregulation of one gene (*SOD2*; 4-fold) at 8 h and 111 gene changes at 24 h, including a 5-fold increase in *SOD2*. Increased levels of SOD2 mRNA at 24 h were also confirmed in HKNM-2 normal human pleural mesothelial cells by qRT-PCR. SOD2 protein levels were increased at toxic concentrations (75×10^6 ^μm^2^/cm^2^) of Libby six-mix at 24 h. In addition, levels of copper-zinc superoxide dismutase (Cu/ZnSOD; SOD1) protein were increased at 24 h in all mineral groups. A dose-related increase in SOD2 activity was observed, although total SOD activity remained unchanged. Dichlorodihydrofluorescein diacetate (DCFDA) fluorescence staining and flow cytometry revealed a dose- and time-dependent increase in reactive oxygen species (ROS) production by LP9/TERT-1 cells exposed to Libby six-mix. Both Libby six-mix and crocidolite asbestos at 75×10^6 ^μm^2^/cm^2 ^caused transient decreases (p < 0.05) in GSH for up to 24 h and increases in gene expression of heme oxygenase 1 (*HO-1*) in LP9/TERT-1 and HKNM-2 cells.

**Conclusions:**

Libby six-mix causes multiple gene expression changes in LP9/TERT-1 human mesothelial cells, as well as increases in SOD2, increased production of oxidants, and transient decreases in intracellular GSH. These events are not observed at equal surface area concentrations of nontoxic glass beads. Results support a mechanistic basis for the importance of SOD2 in proliferation and apoptosis of mesothelial cells and its potential use as a biomarker of early responses to mesotheliomagenic minerals.

## Background

Asbestos is a commercial designation for a group of six mineral fibers that have been used in commerce and industry for decades [1]. Although asbestos is no longer used in building materials in the United States, health hazards associated with various types of asbestos, especially amphibole types that give rise to the devastating cancer malignant mesothelioma (MM), remain a major concern in many countries [2]. Substantial quantities of commercial asbestos and other minerals, such as vermiculite that contains trace amounts of an amphibole fiber, remain in waste piles and buildings at several sites. The mine in Libby, Montana is of particular interest given that at one time it produced up to 80% of the world's supply of vermiculite [3], and exposure occurred outside of Libby at numerous processing plants throughout the United States [4]. Additionally, it is estimated that nearly 1 million homes in the United States have expanded vermiculite-based insulation [5]. Although technically not classified as one of the six types of asbestos, exposure of residents and past workers at the vermiculite mine to Libby six-mix has been associated with the development of pleural plaques [6] and numerous asbestos-related diseases including asbestosis, pleural fibrosis and MMs, respectively [7-11]. In fact, standardized mortality rates from asbestosis in this region, including those of miners, are reported to be 40 to 80 times greater than expected when compared to the reference populations in Montana and the United States, respectively [12]. Lung cancer mortality is also elevated in these individuals compared to the remainder of the United States [13].

The specific mechanisms whereby asbestos causes cellular injury are not completely understood, although they are believed to involve the generation of reactive oxygen species (ROS) from cells or from reduction-oxidation reactions occurring on the surface of high iron-containing fibers (reviewed in [14]). It is unclear whether Libby six-mix has the same molecular and pathogenic effects on cells of the lung and pleura as do amphibole types of asbestos such as crocidolite. Here we used gene expression profiling to define early molecular events occurring in the human mesothelial cell line, LP9/TERT-1, that may contribute to the toxicity of Libby six-mix. Our laboratory has recently utilized this approach to examine transcriptional alterations in LP9/TERT-1 cells following exposure to crocidolite asbestos, nonfibrous talc, fine titanium dioxide (TiO2), or glass beads [15,16]. Our ongoing hypothesis for both the previously reported studies and those discussed here is that the number and magnitude of significant gene changes elicited by minerals correlate with their toxicity and pathogenic potential [15,16]. Results of the current studies in LP9/TERT-1 cells appear to support this hypothesis.

Since microarray analyses following exposure to Libby six-mix showed that only *SOD2 *was upregulated at both time points tested, an observation we confirmed in isolates of HKNM-2 normal human pleural mesothelial cells, we conducted a series of experiments to examine both the potential of this amphibole to generate oxidative stress in LP9/TERT-1 cells, and the functional significance of these changes. This was especially relevant since oxidants generated by asbestos fibers or cells after contact with or uptake of asbestos are linked to toxic and biological manifestations in progenitor and effector cells of asbestos-related diseases [14]. Immunoblotting and activity assays confirmed this increase in SOD2 levels, and DCFDA fluorescence staining and flow cytometry revealed a dose- and time-dependent increase in ROS production by LP9/TERT-1 cells exposed to Libby six-mix. Given the role glutathione (GSH) plays as an antioxidant and signaling molecule in the lung, we examined the effects that Libby six-mix and crocidolite asbestos had on GSH and showed that at a toxic concentration (75×10^6 ^μm^2^/cm^2^), these minerals caused transient decreases in GSH for up to 24 h. These studies are the first to examine the ability of Libby six-mix to elicit transcriptional changes and oxidative stress in human mesothelial cells. These early molecular events may contribute to the toxicity and pre-neoplastic effects of this amphibole in the development of mesotheliomas.

## Results

### Characterization and toxicity of mineral preparations

**Figure 1:**
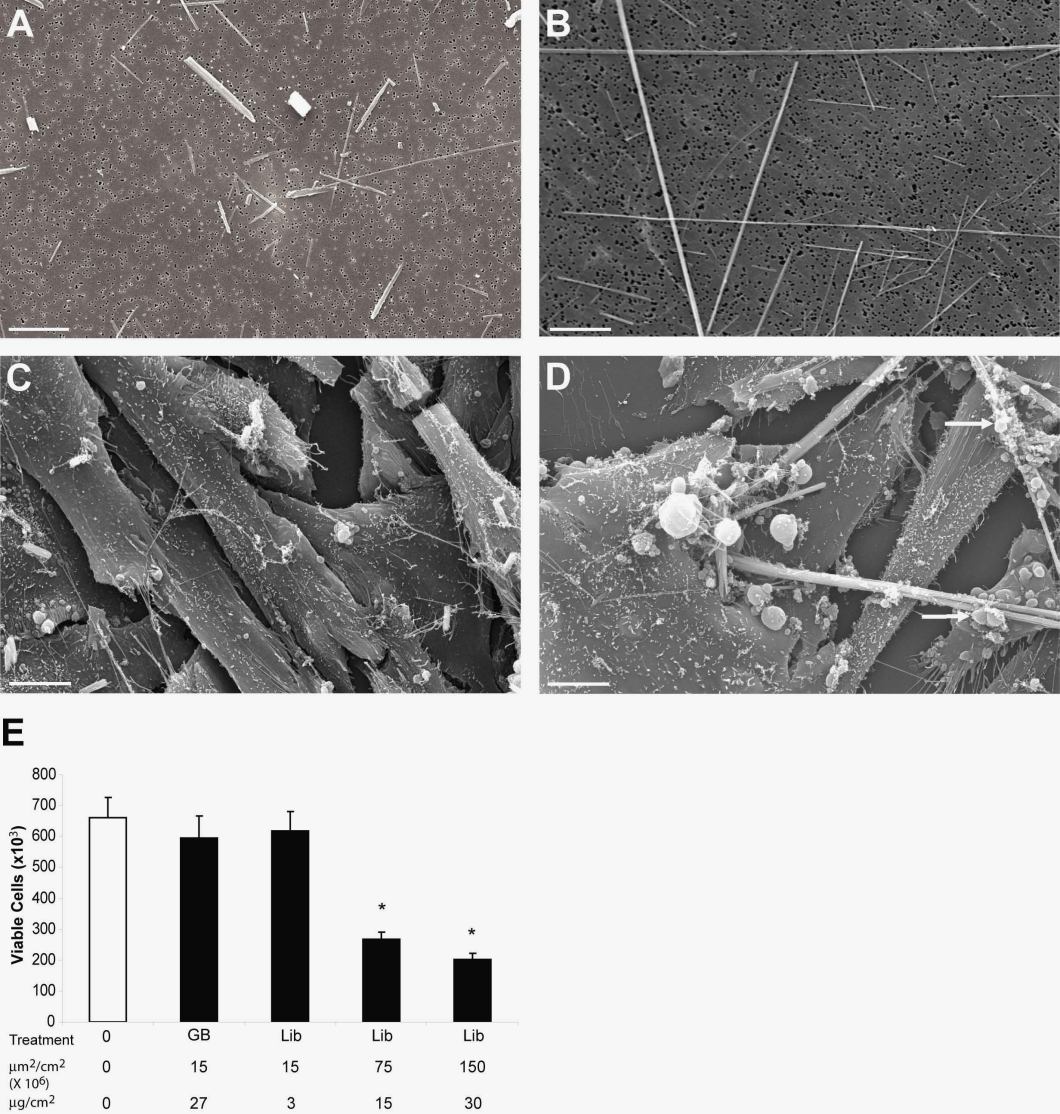
**Interaction of Libby six-mix with LP9/TERT-1 cells and resultant changes in cell viability**. SEM images of (**A**) Libby six-mix, (**B**) crocidolite asbestos, (**C**) LP9/TERT-1 cells alone, and (**D**) LP9/TERT-1 cells interacting with 75×10^6 ^μm^2^/cm^2 ^Libby six-mix. White arrows indicate cell membrane blebbing and exudate formation. All magnifications X 1500, scale bars = 10 μm. (**E**) Viability of LP9/TERT-1 cells following 24 h of exposure to glass beads (non-pathogenic control) or Libby six-mix as determined using 0.4% trypan blue staining of cells detached with Accutase. Bars denote the mean ± SEM of 3 individual experiments where n = 3 replicates per group per experiment. * p < 0.05 compared to glass beads.

Chemical composition, mean surface area (S.A.), and mean size of the glass beads, Libby six-mix and NIEHS crocidolite preparations used in our studies are provided in **Table **1 and have been characterized by others [17-20]. The sample of Libby amphibole we used in the studies described here is often referred to as "six-mix" since it includes six different samples collected at the former mine site, and is comprised of a combination of several amphiboles including winchite, richterite and tremolite (approximately 84%, 11%, and 6% of the respirable fraction, respectively), as well as other trace elements not classified in this mineral family [19]. It possesses diverse morphologies including both asbestiform and non-asbestiform structures (cleavage fragments) exhibiting a wide range of aspect ratios [21]. Trace amounts of other elements occur in NIEHS asbestos standards [22] as well as in Libby six-mix [19], and the fiber size, length and diameter distributions and proportions of cleavage fragments and nonfibrous particles are different between these preparations. For example, blocky particles and small fragments were a feature of the Libby six-mix preparation when examined by SEM (**Figure **1A) as compared to fibrous crocidolite asbestos (**Figure **1B). In addition, the morphology and cellular interactions of Libby six-mix with LP9/TERT-1 cells were examined using SEM. LP9/TERT-1 cells have prominent microvilli and are squamous and contiguous, resembling the normal morphology of mesothelial cells *in vivo *(**Figure **1C). However, treatment with 75×10^6 ^μm^2^/cm^2 ^of Libby six-mix caused contraction of the cells around long fibers, cell membrane blebbing, and exudate formation around fibers (**Figure **1D). Trypan blue exclusion viability studies demonstrated a dose-related increase in cytotoxicity with increasing concentrations of Libby six-mix, but not glass beads (**Figure **1E). Based on these viability studies, we chose the non-toxic Libby six-mix dose of 15×10^6 ^μm^2^/cm^2 ^with which to carry out microarray studies to avoid assaying dead or dying cells. In contrast, higher surface area concentrations (75×10^6 ^μm^2^/cm^2^) of Libby six-mix and crocidolite asbestos caused approximately 60 and 50-80% decreases [16], respectively, in cell viability in LP9/TERT-1 cells at 24 h.

**Table 1:** Mineral characterization

Name	Chemical Composition	Mean S.A. (m^2^/g)^a^	Mean Size (μm)^b^	Source
Glass beads	SiO_2_	3	2.06	Polysciences Inc.
Libby six-mix	See references [18-20]	5	7.21×0.61^c^	USGS
Crocidolite	Na_2_Fe^2+^_3_Fe^3+^_2_Si_8_O_22_(OH)_2_	15	7.40×0.25	NIEHS Reference Sample

^a ^S.A. = surface area.

^b ^Length × diameter for Libby six-mix and crocidolite asbestos, and diameter for glass beads.

^c ^See reference [17].

### Time-related changes in gene expression are observed following exposure of LP9/TERT-1 cells to Libby six-mix

GeneChip^® ^Human Genome U133A 2.0 arrays targeting 18,400 human transcripts were used for microarray analyses of LP9/TERT-1 cells treated with 15×10^6 ^μm^2^/cm^2 ^Libby six-mix for 8 or 24 h. Exposure to Libby six-mix elicited upregulation of only one gene, *SOD2*, at 8 h, and 111 gene changes at 24 h. The top 10 genes upregulated or downregulated by Libby six-mix at 8 and 24 h are listed in **Table **2. No significant (p < 0.05) alterations in gene expression were observed following exposure to glass beads (non-pathogenic control; 75×10^6 ^μm^2^/cm^2^) at either time point (data not shown). TaqMan^® ^quantitative real time PCR (qRT-PCR) was employed to validate increases in *ATF3*, *IL8*, and *SOD2 *gene expression in HKNM-2 normal human pleural mesothelial cells. Also, since *ATF3 *and *IL8 *mRNA expression was increased in LP9/TERT-1 cells treated with 15×10^6 ^μm^2^/cm^2 ^Libby six-mix and in previous studies using crocidolite asbestos [16], qRT-PCR was used to verify these changes (**Table **2). GeneSifter software was utilized to determine the ontology of genes upregulated and downregulated in LP9/TERT-1 cells at 24 h. Overall, 74 genes were upregulated and 37 genes were downregulated at 24 h following exposure to 15×10^6 ^μm^2^/cm^2 ^of Libby six-mix (**Table **3).

**Table 2:** Top 10 gene expression alterations by Libby six-mix (15×10^6 ^μm^2^/cm^2^) in LP9/TERT-1 and HKNM-2 mesothelial cells

Gene Name (Abbreviation)	Fold Change^a^		LP9/TERT-1 qRT-PCR Validation	HKNM-2 qRT-PCR Results^b^
Increased:	8 h	24 h		
Tissue factor pathway inhibitor-2 (TFPI2)	NC	11*	**--**	**--**
Interleukin 8 C-terminal variant, 211506_s_t (IL8)	NC	9*	**--**	3
Prostaglandin-endoperoxide synthase 2 (PTGS2)	NC	7*	**--**	**--**
Interleukin 8 (IL8)	NC	7*	14*	**--**
Pyruvate dehydrogenase kinase, isozyme 4 (PDK4)	NC	7*	**--**	**--**
Pleckstrin homology-like domain, family A, member 1 (PHLDA1)	NC	5*	**--**	**--**
Chemokine (C-X-C motif) ligand 3 (CXCL3)	NC	5	**--**	**--**
Activating transcription factor 3 (ATF3)	NC	5*	5*	5
Superoxide dismutase 2, mitochondrial (SOD2)	4*	5*	**--**	2
Annexin 14 (ANX14)	NC	4	**--**	**--**
				**--**
**Decreased:**				**--**
Oxytocin receptor (OXTR)	NC	5*	**--**	**--**
Chromosome 5 open reading frame 13 (C5orf13)	NC	4*	**--**	**--**
Chromosome 21 open reading frame 7 (C21orf7)	NC	3*	**--**	**--**
Cytochrome P450, family 24, subfamily A, polypeptide 1 (CYP24A1)	NC	3*	**--**	**--**
Calponin 1, basic, smooth muscle (CNN1)	NC	3	**--**	**--**
Cyclin-dependent kinase inhibitor 1C (CDKN1C)	NC	3	**--**	**--**
Methyltransferase like 7A (METTL7A)	NC	2*	**--**	**--**
Periplakin (PPL)	NC	2*	**--**	**--**
Kelch-like 4 (KLHL4)	NC	2	**--**	**--**
Phospholipase C-like 1 (PLCL1)	NC	2*	**--**	**--**

^a ^NC = No significant (p < 0.05) change greater than 2-fold from untreated controls across all 3 replicates.

^b^HKNM-2 normal human pleural mesothelial cells were treated with 15×10^6 ^μm^2^/cm^2 ^Libby asbestos for 24 h.

*Gene in the top 10 genes upregulated/downregulated by crocidolite asbestos at the same time and concentration [16].

**Table 3:** Ontological classifications of gene expression alterations by Libby six-mix (15×10^6 ^μm^2^/cm^2^) in LP9/TERT-1 human mesothelial cells at 24 h

Ontological Classification	No. Genes Upregulated^a^	No. Genes Downregulated
Signal transduction	17	10
Immune response	8	1
Protein metabolic process	7	5
Apoptosis regulation	4	4
Cell proliferation	8	2
Extracellular matrix	2	1
Cell adhesion	2	2
Cell motility	2	0
Oxygen & ROS metabolic process	1	0

^a ^Overall, 74 genes were upregulated and 37 genes were downregulated ( > 2-fold compared to untreated controls; p < 0.05), although only the most common ontological classifications are given.

### SOD2 protein levels and activity are increased in LP9/TERT-1 cells following Libby six-mix exposure

**Figure 2:**
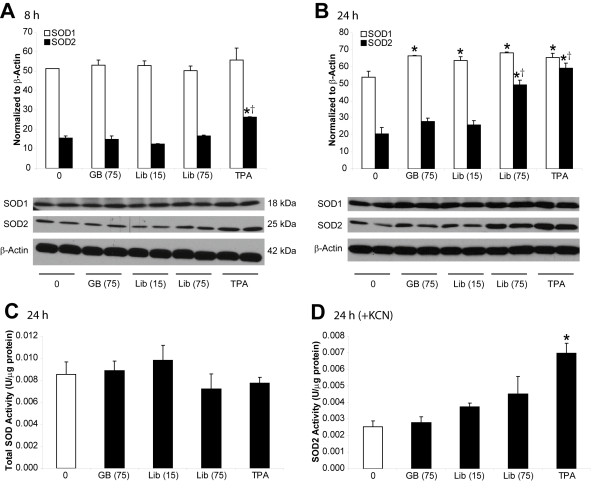
**Effects of Libby six-mix exposure on SOD levels as demonstrated by Western blots and activity assays**. Immunoblotting of SOD1 and SOD2 protein levels using LP9/TERT-1 cell lysates collected following (**A**) 8 h or (**B**) 24 h exposures to glass beads (non-pathogenic control), Libby six-mix, or 100 ng/ml TPA (positive control). Surface area (x10^6 ^μm^2^/cm^2^) is represented in parentheses following mineral name. A total of 30 μg of protein was separated by 10% SDS-PAGE, transferred to nitrocellulose, and immunoblotted using specific SOD1, SOD2, and β-Actin primary antibodies. Quantitative densitometry was performed using QuantityOne software, and values were normalized to β-Actin protein levels. Bars denote the mean ± SEM of n = 2 samples per group and data are representative of 3 separate experiments. * p < 0.05 compared to medium control (0). † p < 0.05 compared to glass beads. (**C**) Total SOD and (**D**) SOD2 activity in LP9/TERT-1 cells following exposure to glass beads, Libby six-mix, or 100 ng/mL TPA for 24 h as determined using the Cayman Superoxide Dismutase Assay Kit. Addition of 3 mM KCN (inhibitor of SOD1 and SOD3) allowed SOD2 activity to be assayed specifically. Total protein concentrations were determined using the Bio-Rad Protein Assay so that final SOD activities could be represented as Units/μg protein. Bars denote the mean ± SEM of n = 3 samples per group, and data are representative of 3 separate experiments. * p < 0.05 compared to all other groups.

Since *SOD2 *gene expression was upregulated at both 8 h (4-fold) and 24 h (5-fold), we further examined the expression of SOD protein and enzyme activity using Western blot analysis on whole-cell lysates from LP9/TERT-1 cells exposed to glass beads (non-pathogenic control; 75×10^6 ^μm^2^/cm^2^), low and high concentrations of Libby six-mix (15 and 75×10^6 ^μm^2^/cm^2^, respectively), and the phorbol ester, TPA (100 ng/mL). At 8 h, there were no significant (p < 0.05) differences observed in SOD1 protein levels, and an increase in SOD2 protein was observed only in the TPA positive control treatment group (**Figure **2A). At 24 h, SOD1 protein levels were significantly (p < 0.05) higher in all treatment groups compared to the medium control group, and SOD2 protein levels were increased in high concentration Libby six-mix (75×10^6 ^μm^2^/cm^2^) and TPA treatment groups (**Figure **2B). Both total SOD and SOD2 activity were then examined at 24 h using an assay employing a tetrazolium salt which, upon reduction with superoxide anions generated via xanthine oxidase activity, forms a formazan dye. Since SOD is capable of catalyzing the reduction of superoxide anions to H_2_O_2_, this assay measures any changes in formazan dye formation that occurs as a result of increased/decreased SOD production following exposure to the minerals of interest. Total SOD activity was unaffected by exposure to Libby six-mix (**Figure **2C). When the SOD1 and SOD3 inhibitor KCN was added to the reactions, a dose related increase in SOD2 activity by Libby six-mix was suggested although insignificant statistically (**Figure **2D).

### DCFDA assays demonstrate increased ROS production in LP9/TERT-1 cells following Libby six-mix exposure

**Figure 3:**
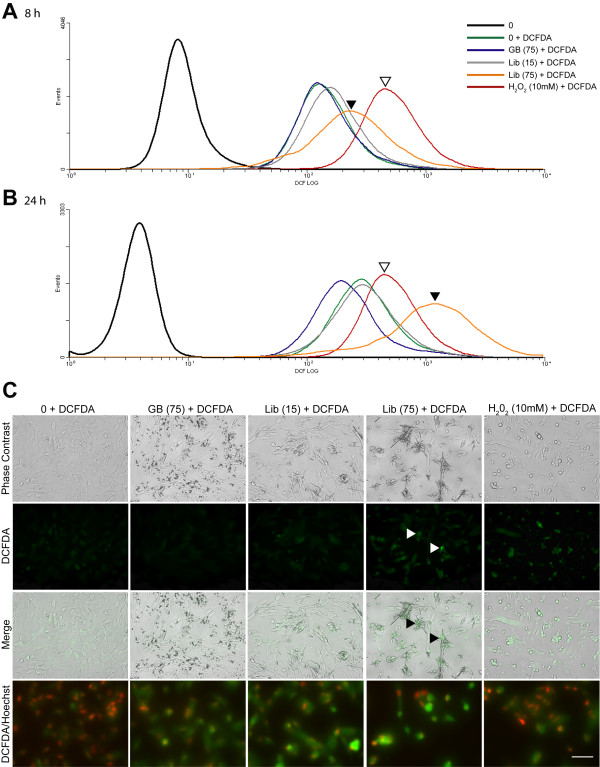
**Determination of ROS generation via detection of DCFDA by flow cytometry and fluorescent microscopy**. ROS generation in LP9/TERT-1 cells following (**A**) 8 h or (**B**) 24 h exposures to glass beads (non-pathogenic control), Libby six-mix, or 10 mM H_2_O_2 _(positive control) as determined by flow cytometric detection of DCFDA staining. White and black arrowheads indicate log shifts following exposure to H_2_O_2 _(10 mM; 20 min) and Libby six-mix (75×10^6 ^μm^2^/cm^2^), respectively. (**C**) DCFDA levels were also visualized using fluorescence microscopy. White arrowheads indicate increased DCFDA levels in Libby six-mix (75×10^6 ^μm^2^/cm^2^) treated cells. In the merged image, black arrowheads indicate identical areas defined by the white arrowheads, and demonstrate that the highest levels of ROS are produced in areas were Libby six-mix contacts LP9/TERT-1 cells. Surface area (x10^6 ^μm^2^/cm^2^) is represented in parentheses following mineral name. Bottom panel shows co-fluorescence of Hoechst dye (red nuclear stain) and DCFDA. Scale bar = 50 μm.

CM-H_2_DCFDA (DCFDA) is a cell permeable, fluorogenic probe that serves as an indirect indicator of intracellular ROS levels. Upon entering the cell, diacetate groups present on CM-H_2_DCFDA are cleaved by intracellular esterases, resulting in a reduced intermediate that can subsequently be oxidized (and thus fluoresces) in the presence of ROS. To confirm that Libby six-mix increased the production of intracellular ROS in LP9/TERT-1 cells, this DCFDA probe was administered to cells following treatment and detected via flow cytometry or fluorescence microscopy. Flow cytometric analysis of LP9/TERT-1 cells exposed to Libby six-mix at 15×10^6 ^or 75×10^6 ^μm^2^/cm^2 ^for 8 and 24 h demonstrated a dose-dependent increase in relative fluorescence indicated by a log shift right (x-axis) in these line histograms (**Figure **3A, **3B**). Both cells in medium without DCFDA and cells in medium with DCFDA were included as controls. Exposure to non-pathogenic glass beads at 75×10^6 ^μm^2^/cm^2 ^had no effect on intracellular ROS production, as line histograms for this treatment group were at, or to the left of, those for the untreated control. Cells exposed to 10 mM H_2_O_2 _for 20 min (positive control) exhibited consistently high ROS levels at both 8 and 24 h.

Fluorescence microscopy confirmed results obtained from flow cytometry analysis (**Figure **3C). Specifically, the 75×10^6 ^μm^2^/cm^2 ^Libby six-mix treatment group possessed a greater number of cells strongly positive for DCFDA staining compared to the medium control, glass beads, or Libby six-mix (15×10^6 ^μm^2^/cm^2^) groups. Phase contrast images revealed cell piling and contraction around Libby six-mix fibers and fragments, whereas exposure to glass beads had little effect on cell morphology. When these phase contrast images were merged with images showing DCFDA fluorescence, peak levels of staining occurred in cells directly in contact with Libby six-mix fibers. A combination of DCFDA (green) and Hoechst nuclear staining (red) was then used to determine if DCFDA staining was localized to small apoptotic nuclei. As can be seen in the bottom panels of **Figure **3C, DCFDA staining was not localized exclusively to cells with apoptotic nuclei in the Libby asbestos and H_2_O_2 _groups.

### Libby six-mix and crocidolite asbestos alter mRNA levels of heme oxygenase 1 (*HO-1*) in LP9/TERT-1 and HKNM-2 cells

**Figure 4:**
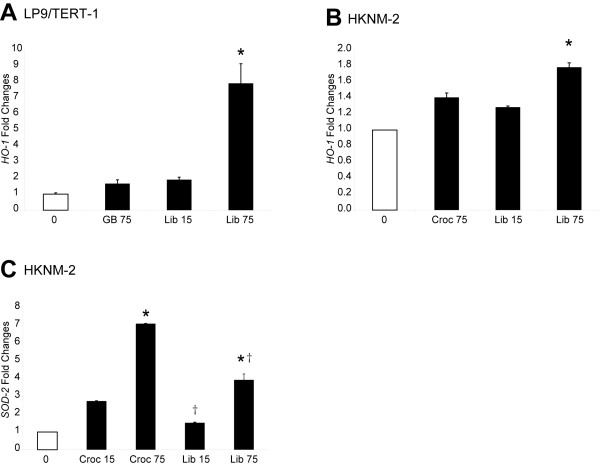
**Gene expression of oxidative stress markers, *HO-1 *in LP9/TERT-1 cells and *HO-1 *and *SOD2 *in HKNM-2 cells**. *HO-1 *expression in (**A**) LP9/TERT-1 cells following exposure to 15 or 75×10^6 ^μm^2^/cm^2 ^Libby six-mix or glass beads (GB) for 24 h. * p < 0.05 in comparison to all other groups. (**B**) *HO-1 *expression in HKNM-2 cells following exposure to 15 or 75×10^6 ^μm^2^/cm^2 ^Libby six-mix or crocidolite asbestos for 24 h. * p < 0.05 in comparison to all other particle-exposed groups. (**C**) SOD2 expression in HKNM-2 cells following exposure to 15 or 75×10^6 ^μm^2^/cm^2 ^crocidolite asbestos or Libby six-mix for 24 h. * p < 0.05 in comparison to lower concentration of respective fiber. † p < 0.05 in comparison to respective concentrations of crocidolite asbestos. Bars denote the mean ± SEM of n = 2 or 3 samples per group per experiment.

In order to further support the concept that Libby six-mix and crocidolite asbestos elicit oxidative stress in LP9/TERT-1 cells, we evaluated changes in heme oxygenase 1 (*HO-1*) gene expression [23] 24 h following treatment with 15 or 75×10^6 ^μm^2^/cm^2 ^Libby six-mix. Libby six-mix caused a significant upregulation of *HO-1 *at 75×10^6 ^μm^2^/cm^2 ^in LP9/TERT-1 cells that was not observed with identical concentrations of glass beads (GB) or at lower concentrations of Libby six-mix (**Figure **4A). Although few numbers of HKNM-2 normal human pleural mesothelial cells precluded us from performing duplicate untreated control samples, the HKNM-2 cells responded similarly to LP9/TERT-1 cells in that dose-related responses were observed after exposures to Libby amphibole (**Figure **4B). These results indicate that LP9/TERT-1 cells are an acceptable cell line in that they mimic normal human mesothelial cells in their responses to minerals. This fact was further substantiated by data showing that *SOD2 *expression in HKNM-2 cells was also significantly increased following exposure to 75×10^6 ^μm^2^/cm^2 ^of crocidolite asbestos or Libby six-mix for 24 h (**Figure **4C and **Table **2).

### Libby six-mix and crocidolite asbestos deplete levels of reduced glutathione (GSH) in LP9/TERT-1 cells

**Figure 5:**
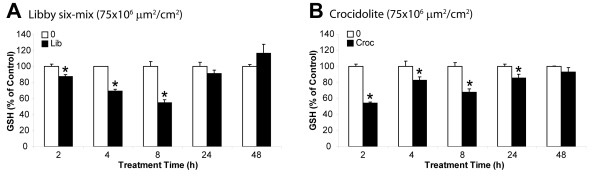
**Effects of Libby six-mix exposure on glutathione levels in LP9/TERT-1 cells**. Glutathione (GSH) levels in LP9/TERT-1 cells following exposure to (**A**) Libby six-mix (75×10^6 ^μm^2^/cm^2^) or (**B**) crocidolite asbestos (75×10^6 ^μm^2^/cm^2^) as measured via HPLC over a 48 h period. GSH levels are defined as percent of the medium control (0) at the corresponding time point. Bars denote the mean ± SEM of n = 3 samples per group, and data are representative of 2 separate experiments. * p < 0.05 compared to medium control (0).

Depletion of GSH is generally considered an indicator of oxidative stress. Since it has been demonstrated previously that crocidolite asbestos depletes intracellular GSH levels in C10 murine type II epithelial cells [24], it was included in these time course studies as a positive control. Levels of GSH in LP9/TERT-1 cells were determined by HPLC at specific time points, and data showed that exposure to Libby six-mix caused a preliminary decrease in GSH from 2 to 8 h, followed by a progressive recovery in GSH levels through 48 h (**Figure **5A). Exposure to crocidolite resulted in significant GSH depletion at all time points prior to and including 24 h (**Figure **5B).

## Discussion

Our studies are the first to use robust gene profiling to characterize alterations in gene expression in human mesothelial cells after exposure to Libby six-mix. Prior to initiating microarray studies, we determined a concentration of Libby six-mix that was not overtly toxic to LP9/TERT-1 cells to avoid the induction of gene expression secondary to cell death. This was accomplished by performing dose-response cell viability studies where LP9/TERT-1 cells were exposed to 15×10^6^, 75×10^6^, and 159×10^6 ^μm^2^/cm^2 ^Libby six-mix for 24 h. Results demonstrated that, similar to what we have reported previously for crocidolite asbestos [16], Libby six-mix caused decreases in cell viability at 24 h that were significant at 75×10^6 ^μm^2^/cm^2 ^and associated with cell membrane blebbing, contraction of the cells, and exudate formation around long fibers. Based upon our toxicity data, the low (15×10^6 ^μm^2^/cm^2^), nontoxic concentration of Libby six-mix and comparable surface areas of crocidolite and glass beads were chosen for microarray studies. When the top 10 gene changes following Libby six-mix exposure were compared to those reported for LP9/TERT-1 cells following crocidolite asbestos exposure at identical surface area concentrations [16], numerous similarities were observed. Libby six-mix induced significant upregulation of only one gene at 8 h (*SOD2*, 4-fold). In contrast, *SOD2 *(6-fold increase) was one of many genes upregulated at 8 h following crocidolite asbestos exposure [16]. Of the top 10 genes upregulated at 24 h in response to Libby six-mix, 8 were identical to those reported at 24 h following crocidolite asbestos exposure (*TFPI2*, *IL8 C-terminal variant*, *IL8*, *PTGS2*, *PDK4*, *PHLDA1*, *ATF3*, and *SOD2*). Similarly, 7 of the top 10 genes downregulated at 24 h in response to Libby six-mix were identical to those reported for crocidolite asbestos (*OXTR*, *C5orf13*, *C21orf7*, *CYP24A1*, *METTL7A*, *PPL*, and *PLCL1*). Gene ontology classifications for Libby six-mix also exhibited patterns similar to crocidolite asbestos whereby the most changes in expression (either upregulated or downregulated) occurred in genes associated with signal transduction, protein metabolism, cell proliferation, and immune responses [16].

Several genes upregulated in LP9/TERT-1 cells exposed to Libby six-mix or crocidolite asbestos are associated with inflammation, including the pro-inflammatory cytokine interleukin-8 (*IL8*). This cytokine is chemotactic for polymorphonuclear neutrophils (PMNs) and can subsequently serve as an activator for these inflammatory cells [25]. *IL8 *is produced by a wide array of cells including mesothelial cells, and pleural inflammation is known to be initiated by *IL8 *secretion in response to asbestos exposure [26,27]. Several studies looking at serum *IL8 *levels in workers occupationally exposed to asbestos revealed significant increases in this cytokine [28,29]. Activating transcription factor 3 (ATF3), a member of the cAMP-responsive element-binding (CREB) transcription factor family encoding two isoforms leading to repression or activation of genes, was also upregulated by both Libby six-mix and crocidolite asbestos. Previous work in our laboratory has shown that silencing ATF3 in LP9/TERT-1 mesothelial cells can consequently modulate the production of several well-known asbestos-induced inflammatory cytokines and growth factors including IL-1β, IL-13, granulocyte colony-stimulating factor (G-CSF), vascular endothelial growth factor (VEGF), and platelet-derived growth factor-BB (PDGF-BB) [16]. Prostaglandin-endoperoxide synthase 2 (*PTGS2*; also known as cyclooxygenase 2 or *COX2*) is an integral enzyme in the biosynthesis of prostenoids, and is implicated in carcinogenesis by modulating inflammation, mitogenesis, cell adhesion, and apoptosis. Exposure of a monocyte-macrophage (J774) cell line to an asbestos-like amphibole fiber known as fluoro-edenite induced a significant increase in *PTGS2 *expression [30], and immunohistochemical characterization of human MM samples demonstrated that PTGS2 was highly expressed in these tissues, but not in nonreactive mesothelial tissues from the same individuals [31]. Based upon the upregulation of these genes and others, inflammation induced in an autocrine/paracrine fashion by mesothelial cells may play a critical role in Libby six-mix and crocidolite asbestos-induced cell injury and disease.

In our studies, superoxide dismutase 2 (*SOD2*; manganese superoxide dismutase), a mitochondrial antioxidant protein which catalyzes the dismutation of superoxide (O_2_^•-^) to hydrogen peroxide (H_2_O_2_), was the only gene significantly upregulated by Libby six-mix and crocidolite asbestos at both 8 and 24 h. This increased expression of *SOD2 *recapitulates previously reported data showing elevated *SOD2 *expression/activity in human pleural mesothelial cells or tracheal epithelial cells exposed to crocidolite or chrysotile asbestos [23,32]. The fact that *SOD2 *expression is upregulated following Libby six-mix and crocidolite or chrysotile asbestos exposures [23,32], likely represents activation of a defense mechanism to counteract oxidative stress induced by these fibers, specifically through the dismutation of O_2_^•- ^to H_2_O_2_. Increases in steady-state levels of H_2_O_2 _can subsequently lead to increases in cell proliferation, invasion, migration, metastasis, and resistance to apoptosis [33-35]. In addition, we have previously shown that transfection of *SOD2 *into rodent tracheal epithelial cells ameliorates crocidolite asbestos-induced toxicity, suggesting a role of this gene in cell survival from asbestos [36].

Elevated *SOD2 *levels have been observed in rodent lungs after inhalation of crocidolite asbestos [37,38] and in several cancer cell types including gastric [39], colorectal [40], breast [41], and MM [42]. Based upon strong SOD2 immunoreactivity in MMs in contrast to adenocarcinomas [43], and low SOD2 levels in healthy human pleural mesothelium compared to high endogenous levels in MM lines [44], SOD2 has been proposed as a diagnostic marker for MM. Moreover, polymorphisms of glutathione-*S*-transferase M1 (*GSTM1*) and *SOD2 *are associated with increased risk of MM, findings contributing to the hypothesis that imbalances between oxidative stress and antioxidant enzymes are features of the pathogenesis of MM [45]. Our results showing that early increases in expression, protein levels, and activity of *SOD2 *occur in human mesothelial cells after exposure to MM-inducing fibers therefore may be valuable in designing predictive assays for fiber pathogenicity.

Several studies have been conducted recently examining the effects of Libby six-mix and crocidolite asbestos in tandem. For example, gene expression studies in lungs of C57Bl/6 mice instilled intratracheally with Libby six-mix or crocidolite asbestos revealed common gene ontologies related to the plasma membrane, transport channels and signal transduction [46]. Lung fibrosis and collagen deposition were also observed in mice 6 months following exposure to either Libby six-mix or crocidolite asbestos, although the extent of changes observed in Libby six-mix exposed mice was consistently less [46]. A second C57Bl/6 mouse study demonstrated that at 1 week, 1 month, and 3 months post intratracheal instillation of Libby six-mix or crocidolite asbestos at equal weight concentrations, both amphiboles increased the gene expression of Col1A1, Col1A2, and Col1A3, collagen protein deposition, and inflammation [47]. Again, crocidolite asbestos induced a greater response in the majority of these endpoints when compared to Libby six-mix. However, in both these experiments, an equal mass of Libby six-mix was compared to crocidolite asbestos, possibly reflecting lower fiber numbers or different length and diameter ratios of fibers per unit weight.

*In vitro *experiments using a murine macrophage-like cell line (RAW264.7 cells) and alveolar macrophages lavaged from C57Bl/6 mice and exposed to 62.5 μg/cm^2 ^Libby six-mix for up to 3 h also have shown increases in intracellular ROS levels [17]. This response was subsequently linked to increases in O_2_^•- ^by demonstrating that Libby six-mix exposure led to increased dihydroethidine (DHE) fluorescence, a probe known to preferentially detect superoxide [48]. These increases in intracellular ROS in murine macrophages resulted in oxidative DNA damage as indicated by increased relative levels of 8-oxo-dG and the percentage of cells in the sub-G1 phase following exposure to crocidolite asbestos, but not Libby six-mix, at an equal weight concentration. Decreased intracellular GSH levels was also found to be a feature of Libby six-mix and crocidolite asbestos toxicity in this cell type [17]. On the basis of these results, the authors suggest that separate cellular responses are induced by these two minerals *in vitro *[17]. Although it appears from our research that similar mechanisms contribute to the toxicity of these minerals, their overall differences in toxicity and pathogenicity as demonstrated in work cited above, may reflect different numbers, sizes, and proportions of fibers to nonfibrous particles and fragments, as well as the diverse chemical composition of these different amphiboles. In our studies, oxidant generation and GSH depletion occurred only at the highest concentration of Libby six-mix where approximately 60% of cell death occurred at 24 h. The viable cells at this time point may represent those not killed directly by oxidative stress because of intrinsically higher antioxidant defenses or cells exhibiting adaptive responses such as compensatory proliferation in response to this material. DCF fluorescence does not appear to occur coincidentally with cell death as it is often observed in non-apoptotic cells. The molecular parameters governing these responses and their relationship to cell injury and pleural disease demand further examination.

## Conclusions

After addition at nontoxic concentrations to LP9/TERT-1 mesothelial cells, Libby six-mix (15×10^6 ^μm^2^/cm^2 ^for 24 h) caused significant (p < 0.05) changes in 111 genes, whereas crocidolite asbestos at identical surface area concentrations and time points caused significant changes in 205 genes [16]. No significant alterations in gene expression were observed following exposure to glass beads (75×10^6 ^μm^2^/cm^2^) at either time point, establishing this material as an appropriate non-pathogenic control particle. Results from gene profiling studies suggest that the toxicity induced by Libby six-mix and crocidolite may be acting through a similar mechanism of action in LP9/TERT-1 mesothelial cells. Moreover, new data here support our hypothesis that the number and magnitude of significant gene changes following exposure to pathogenic mineral fibers are indicative of their mesotheliomagenicity. Increases in *SOD2 *and *HO-1 *gene expression were associated with increased production of oxidants and transient decreases in intracellular GSH. These results provide a mechanistic basis for the importance of SOD2 in the proliferation and apoptosis of mesothelial cells and its implementation as a potential biomarker of early responses to minerals capable of causing MM.

## Methods

### Human Mesothelial Cell Cultures

Human mesothelial LP9/TERT-1 cells, an hTERT-immortalized cell line phenotypically and functionally resembling normal human mesothelial cells [49], were obtained from Dr. James Rheinwald (Bringham and Women's Hospital, Boston, MA). LP9/TERT-1 cells were maintained in DMEM/F-12 (1:1) medium (Mediatech, Inc., Herndon, VA) supplemented with 10% fetal bovine serum (FBS) (Mediatech), penicillin-streptomycin (50 U/ml penicillin G, 50 μg/ml streptomycin sulfate) (GIBCO, Carlsbad, CA), hydrocortisone (100 μg/ml), insulin (2.5 μg/ml), transferrin (2.5 μg/ml) and selenium (2.5 μg/ml) (Sigma, St. Louis, MO). HKNM-2 normal human pleural mesothelial cells were isolated at autopsy by Dr. Helmut Popper (University of Graz, Austria) and were grown in Optimem/Hams F-12 3:1 containing 20% FBS, EGF (20 ng/ml), insulin (0.5 μg/ml), hydrocortisone (0.4 μg/ml), penicillin (50 U/ml) and streptomycin sulfate (100 μg/ml) (Sigma, St. Louis, MO). Cells at near confluency were switched to maintenance medium containing 0.5% FBS overnight prior to mineral/agent exposure.

### Mineral Characterization

The Libby amphibole used in these studies was obtained from the United States Geological Service (USGS) and has been physically and chemically characterized previously [18-20,50]. This sample is comprised of six different samples collected at the former Libby vermiculite mine site and is therefore termed Libby "six-mix". The physical and chemical characterization of the NIEHS reference sample of crocidolite asbestos has been reported previously as well [51]. The surface area of asbestos fibers and particles was measured using nitrogen gas sorption analysis to allow computation of identical surface area amounts of minerals to be added to cells. Fiber and particle size dimensions were determined by scanning electron microscopy (SEM) as described previously [52]. The chemical composition, surface area, mean size, and source of each mineral preparation are presented in **Table **1.

### Introduction of Minerals/Agents to Cells

Following sterilization under ultraviolet light overnight, minerals were suspended in 1X Hanks' Balanced Salt Solution (HBSS) at 1 mg/ml, sonicated for 15 min in a water bath sonicator, and triturated 5 times through a 22-gauge needle. This suspension was added to cells in medium to achieve the desired surface area-based concentrations. Phorbol 12-myristate 13-acetate (TPA; 100 ng/ml) was used as a positive control for immunoblotting and SOD activity, and hydrogen peroxide (H_2_O_2_; 10 mM for 20 min) was used as a positive control for the DCFDA assay. H_2_O_2 _(Sigma, St. Louis, MO) was added directly to the medium and TPA (Sigma, St. Louis, MO) was dissolved in dimethylsulfoxide (DMSO) prior to addition to the medium.

### Scanning Electron Microscopy (SEM)

For imaging Libby six-mix and crocidolite asbestos alone, 0.0027 or 0.0023 g was diluted to a final concentration 1.35 or 1.15 μg/ml (4.0 ml total volume), respectively, in a solution of 6% ethanol and ddH_2_O by serial dilution. The Libby six-mix dilution was filtered through a 0.4 μm Nucleopore Track-Etch membrane (Fisher Scientific, Pittsburgh, PA) followed by a rinse with 1 ml 100% ethanol and drying overnight. Half of the dried filter was adhered to a standard aluminum specimen stub with colloidal silver paste (Electron Microscope Sciences, Hatfield, PA) followed by sputter coating with gold and palladium using a Polaron sputter coater (Model 5100; Quorum Technologies, East Sussex, UK). Images were acquired using a JEOL 6060 scanning electron microscope (JEOL USA, Inc., Peabody, MA) following a randomized design of field selection to obtain representative images of the sample. In order to determine interactions between cells and Libby six-mix, cells were grown on Thermonox plastic cover slips (Nalgen Nunc International, Naperville, IL), exposed to 75×10^6 ^μm^2^/cm^2 ^Libby six-mix for 24 h, and then processed for SEM as described previously [52].

### Cell Viability

After 24 h, cells were collected with Accutase cell detachment reagent, and final cell suspensions in Accutase complete medium/HBSS were mixed with 0.4% trypan blue stain, a diazo dye which is retained by dead cells and excluded by viable cells. After 5 min, unstained viable cells were counted using a hemocytometer to determine the total number of viable cells per dish. Based on the results of cell viability studies, Libby six-mix was evaluated in LP9/TERT-1 mesothelial cells at both low and high concentrations (15×10^6 ^and 75×10^6 ^μm^2^/cm^2^, respectively) at 8 and 24 h for the majority of assays. However, for gene profiling experiments employing microarrays, Libby six-mix was tested at the non-toxic concentration of 15×10^6 ^μm^2^/cm^2 ^only. Control groups typically included a negative control consisting of cells maintained in medium alone and a control consisting of cells exposed to the non-pathogenic glass beads at surface area concentrations equaling the highest Libby six-mix concentration utilized.

### RNA Preparation and Microarrays

Total RNA was prepared using an RNeasy^® ^Plus Mini Kit according to the manufacturers' protocol (Qiagen, Valencia, CA), as published previously [53]. Microarrays were performed on samples from 3 independent experiments. For each experiment, n = 3 dishes were pooled into one sample per treatment group giving a total of n = 3 RNA samples per group. All procedures were performed by the Vermont Cancer Center DNA facility using a standard Affymetrix protocol as described previously [53,54]. GeneChip^® ^Human Genome U133A 2.0 arrays (Affymetrix, Santa Clara, CA) targeting 18,400 human transcripts were scanned twice (Hewlett-Packard GeneArray Scanner), the images overlaid, and the average intensities of each probe cell compiled. Microarray data were analyzed using GeneSifter software (VizX Labs, Seattle, WA). This program used a *t*-test for pair-wise comparison and a Benjamini-Hochberg test for false discovery rate (FDR 5%) to adjust for multiple comparisons. A 2-fold cutoff limit was used for analysis.

### Quantitative Real Time PCR (qRT-PCR)

Total RNA (1 μg) was reverse-transcribed with random primers using the AMV Reverse Transcriptase kit (Promega, Madison, WI) according to the recommendations of the manufacturer, as described previously [53]. To quantify gene expression, the cDNA was amplified by TaqMan^® ^qRT-PCR using the 7700 Sequence Prism Detector (Perkin Elmer Applied Biosystems, Foster City, CA). Fold change in the genes of interest was calculated using the ΔΔC_t _method. Duplicate assays were performed with RNA samples isolated from at least 3 independent experiments. The values obtained from cDNAs and hypoxanthine phosphoribosyl transferase (*hprt*) controls provided relative gene expression levels for the gene locus investigated. The Assays-On-Demand™ primer and probe sets used for all qRT-PCR experiments were purchased from Applied Biosystems (Foster City, CA).

### Western Blots

Cells were exposed to agents as described above, the medium aspirated, and cells washed three times with ice-cold PBS prior to collection in 4X sample buffer (200 μM Tris, pH 6.8, 4% SDS, 4 mg/ml β-mercaptoethanol, 40% glycerol, 2 μM pyronin-Y). The amount of protein was determined using the RC DC protein assay (Bio-Rad, Hercules, CA). A total of 30 μg of protein was separated by 10% SDS-PAGE and transferred to nitrocellulose. Western blots were performed as described previously [55], using antibodies specific to SOD1 (rabbit polyclonal, 1:1000, 18 kDa molecular weight (MW); Cell Signaling Technology, Danvers, MA), SOD2 (goat polyclonal, 1:200, 25 kDa MW; Santa Cruz Biotechnology, Inc., Santa Cruz, CA), and β-Actin (mouse monoclonal, 1:2000, 42 kDA MW; Abcam, Cambridge, MA). Quantity One^® ^v.4.4.1 software (Bio-Rad) was used to quantify band density, and values were normalized to β-Actin protein levels.

### SOD Activity Assays

Cells were washed three times with ice-cold PBS and collected in 500 μl - 1 ml of cold buffer (20 mM 4-(2-hydroxyethyl)-1-piperazineethanesulfonic acid (HEPES), 1 mM ethylene glycol tetraacetic acid (EGTA), 210 mM mannitol, and 70 mM sucrose, pH 7.2). Cells were then sonicated 5 × 2 sec, centrifuged at 1500 × g at 4°C for 10 min and supernatants removed. These supernatants were subsequently used to conduct the SOD activity assay according to the manufacturer's protocol (Superoxide Dismutase Assay Kit; Cayman Chemical Company, Ann Arbor, MI). In order to assay MnSOD (SOD2) activity specifically, 3 mM potassium cyanide (KCN) was added to the reactions to inhibit both Cu/ZnSOD (SOD1) and extracellular SOD (SOD3). Total protein concentrations in supernatants were also determined using the Bio-Rad Protein Assay so that final SOD activities could be represented as Units/μg protein.

### DCFDA Assays

Following treatment, the cell medium was removed and replaced with DMEM/F-12 (1:1) medium without phenol red (GIBCO, Carlsbad, CA) supplemented as described previously and containing 0.5 μM 5-(and-6)-chloromethyl-2',7'-dichlorodihydrofluorescein diacetate, acetyl ester (CM-H_2_DCFDA; Invitrogen, Carlsbad, CA). Cells were incubated in this medium for 15 min in the dark at 37°C and then trypsinized. Following neutralization with fully supplemented DMEM/F-12 (1:1) medium (without phenol red), cells were collected and spun for 4 min at 1200 rpm at 4°C to generate a cell pellet. This pellet was washed and subsequently resuspended in 800 μl of 1X HBSS without phenol red (GIBCO, Carlsbad, CA). Samples containing asbestos were filtered through 50 μm nylon mesh prior to being read on a Beckman Coulter Epics XL flow cytometer (Beckman Coulter, Inc., Brea, CA). Flow cytometry data was analyzed and histograms generated using WinMDI 2.8 software. For DCFDA visualization studies, treated cells were visualized on an Olympus IX70 inverted microscope immediately following DCFDA exposure to produce either phase contrast or fluorescent images (460-495 nm/510 nm excitation/emission). Fluorescent microscope images were pseudocolored green. To determine whether DCFDA was exclusively localized in cells with shrunken nuclei, i.e., apoptotic or necrotic, cells were incubated at 37°C for 15 min in 1 ug/mL of Hoechst 33342 nuclear stain (Invitrogen, Carlsbad, CA) in DMEM/F-12 (1:1) medium with phenol red containing 0.5% FBS. Cells were then washed in medium and incubated at 37°C for 15 min in medium with 0.5 μM DCFDA in the dark. Nuclear stain (350 nm/461 nm excitation/emission) and DCFDA (460-495 nm/510 nm excitation/emission) were imaged on an Olympus IX70 inverted microscope with the nuclei pseudocolored red and DCFDA pseudocolored green.

### Reduced Glutathione (GSH) Assay

Treated cells were washed three times with ice-cold PBS, incubated on ice in 200 μl of 1X cell lysis buffer (Cell Signaling Technology, Danvers, MA) containing 1 mM PMSF, and collected using a cell lifter. Cells were then centrifuged at 14000 × g at 4°C and supernatants removed and derivitized. Derivitization and high performance liquid chromatography (HPLC) detection of reduced glutathione (GSH) were performed as described previously [24]. The Bio-Rad Protein Assay (Bio-Rad Laboratories, Hercules, CA) was performed on supernatants as well to determine total protein concentration. GSH concentrations were subsequently divided by this protein concentration to obtain nmol GSH/mg protein. Final data are represented as the percent of GSH levels compared to untreated controls at each time point.

### Statistical Analysis

Data from cell viability, western blotting, and SOD activity assays were evaluated by analysis of variance (ANOVA) using the Student Neuman-Keul's procedure for adjustment of multiple pair-wise comparisons between treatment groups. Glutathione depletion studies were analyzed via two-way ANOVA. Differences in gene expression values determined by qRT-PCR were evaluated using a Student's *t*-test. Differences with p values < 0.05 were considered statistically significant.

## Competing interests

The authors declare that they have no competing interests.

## Authors' contributions

JMH and BTM conceived of the overall research plan. MEG provided the Libby six-mix samples. SAL, TNP, and VA conducted SEM. AS and MBM aided in maintaining cell cultures, prepared RNA, and performed viability assays and microarray analysis. AvdV provided the protocol, reagents, and HPLC equipment to conduct the GSH depletion studies. JMH maintained cell cultures, interpreted the data, and performed the following: qRT-PCR, Western blotting, SOD activity assays, DCFDA assays and assays for GSH depletion. PMV performed statistical analyses on data sets. JMH and BTM drafted the manuscript. All authors read and approved the final manuscript.
